# Iron Deposition and Functional Connectivity Differences in Females With Migraine Without Aura: A Comparative Study of Headache Sides

**DOI:** 10.1002/brb3.70096

**Published:** 2024-10-22

**Authors:** Yan Zhang, Mingxian Bai, Zhenliang Xiong, Qin Zhang, Lihui Wang, Xianchun Zeng

**Affiliations:** ^1^ Key Laboratory of Intelligent Medical Image Analysis and Precise Diagnosis of Guizhou Province State Key Laboratory of Public Big Data, College of Computer Science and Technology Guizhou University Guiyang Guizhou China; ^2^ Department of Radiology, International Exemplary Cooperation Base of Precision Imaging for Diagnosis and Treatment Guizhou Provincial People's Hospital Guiyang Guizhou China; ^3^ Guizhou University Medical College Guiyang Guizhou China; ^4^ First School of Clinical Medicine Zunyi Medical University Zunyi Guizhou China

**Keywords:** functional connectivity, iron deposition, laterality, migraine without aura

## Abstract

**Background:**

The pathophysiological mechanisms underlying migraine without aura (MwoA) in females remain incompletely elucidated. Currently, the association between headache laterality and iron deposition (ID), and functional connectivity (FC) in female MwoA patients has not been fully studied.

**Methods:**

We prospectively recruited 63 female patients with MwoA and 31 matched healthy controls (HC) from the hospital. ID and FC among the four groups were analyzed using two‐sample *t*‐tests (with cluster‐wise family‐wise error [FWE] correction). Pearson correlation analysis was used to evaluate the relationships between clinical variables and both ID and FC values. Significance level: *p* < 0.05.

**Results:**

Compared to HC, left‐sided MwoA exhibited differences in ID in various brain regions, including the cerebellum, left orbital inferior frontal gyrus, left calcarine gyrus, right putamen, and left caudate nucleus, as well as exhibited enhanced FC between the left lobule III of the cerebellum and the right superior temporal gyrus. Compared to bilateral MwoA, left‐sided MwoA showed significantly enhanced in FC values in the left calcarine gyrus, the right precentral gyrus, the right postcentral gyrus, and the right lingual gyrus. Additionally, significant differences were observed in the Pearson correlations between clinical variables and both ID and FC in the female MwoA subgroups.

**Conclusion:**

Our study provided preliminary evidence indicating significant differences in ID, FC, and correlations among subgroups of female MwoA. This provides neuroimaging references for further subclassifying MwoA patients. This offers valuable insights into potential pathophysiological mechanisms linked to the brain functional impairment in female MwoA.

## Graphical abstract

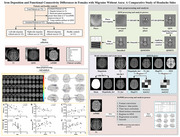

## 1 Introduction

Migraine is a hereditary primary headache disorder (Morillo 2002), and the global burden of disease studies indicates that it has become the second leading cause of global disability (Steiner et al. 2020). Migraine has emerged as a significant public health and social issue due to its high global prevalence, imposing a substantial healthcare burden on patients and their families, resulting in a severe reduction in the quality of life for affected individuals (Li et al. 2020). The International Classification of Headache Disorders, Third Edition (ICHD‐III), identifies two main types of migraine: migraine without aura (MwoA) and migraine with aura (Li et al. 2020). Among these, MwoA is the most common subtype, accounting for approximately 64% of migraine cases (Li et al. 2020). Furthermore, migraines exhibit lateralization (Blum et al. 2023), and based on the predominant side of the headache during the attack, migraine sufferers can be categorized into left‐sided migraines, right‐sided migraines, and bilateral migraines. Studies have found that the lateralization of migraines affects patients differently. For example, left‐sided migraines are associated with more severe anxiety and depression (Cologno et al. 2005). Left‐sided and bilateral headaches have a higher impact on physiological and psychological aspects, including tinnitus, depression, and poorer quality of life (Langguth et al. 2017). Post‐traumatic stress disorder is more common in left‐sided migraine patients (Chakravarty, Mukherjee, and Roy 2008). Additionally, influenced by estrogen, the incidence of migraine is higher in females with a threefold higher prevalence compared to males (Nappi et al. 2022), ranking as the first leading cause of disability in young women, affecting 18% of women (Steiner et al. 2020). Moreover, female migraines have a higher frequency of attacks, longer duration, greater intensity, and more disabling effects (Finocchi and Strada 2014). The unique cortical network characteristics in the female brain may contribute to more extensive brain damage in female migraine patients (Liu et al. 2011). However, the pathophysiological mechanisms of lateralized headaches in female MwoA patients are not fully understood.

Gender is a significant factor influencing the structure and function of the brain. Compared to males, females have a larger gray matter volume in the frontal lobe and parietal lobe (Allen et al. 2003), and thicker cortices in the parietal and temporal lobes (Luders et al. 2006). Additionally, gender‐related differences in brain iron deposition (ID) exist, with females having lower peripheral iron levels than males (Fleming et al. 2001), and lower brain iron levels in structures such as the caudate nucleus, thalamus, frontal white matter, and red nucleus (Bartzokis et al. 2007; Gong et al. 2015). Recent studies suggest that brain iron concentration may impact the functional networks of the brain. For example, changes in iron content in the striatum can affect the consistency of the resting‐state functional networks between the caudate nucleus and putamen and other parts of the brain (Salami et al. 2018). Iron concentration in the substantia nigra can influence functional networks and alter working memory performance in young individuals (Xu et al. 2021). Elevated cortical iron concentration can disrupt functional networks supporting working memory in older individuals (Zachariou et al. 2020). Previous research has utilized resting‐state functional magnetic resonance imaging (rsfMRI) to analyze functional connectivity (FC) in migraine patients (Salami et al. 2018; Tomasi and Volkow 2012; Biswal et al. 2010), and some studies have used quantitative susceptibility mapping (QSM) techniques to explore ID in migraine patients (Gong et al. 2015; Xu et al. 2021; Zachariou et al. 2020). Wang et al. (2023) conducted a comparative analysis of 1444 female patients with MwoA based on migraine and its related factors (such as side, location, and frequency), concluding that patients with MwoA should be considered for further subdivision into subgroups. However, current research is susceptible to interference from male patients, and research on laterality, ID, and FC in female patients with MwoA is not in‐depth enough.

In this study, we utilized rsfMRI and QSM data, based on the automated anatomical labeling (AAL) brain atlas (Rolls et al. 2020), and data‐driven analytical methods to explore the differences in ID and FC among female patients with MwoA with different headache sides. Additionally, we calculated the Pearson correlation between clinical variables and both ID and FC.

## 2 Materials and Methods

### 2.1 Subjects

**FIGURE 1:**
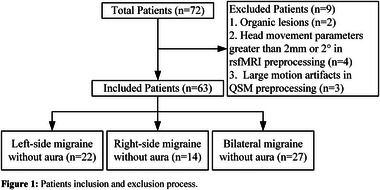
Patients inclusion and exclusion process.

Prospectively recruited 72 female patients with MwoA and 31 matched healthy controls (HC) from the hospital outpatient department. Excluding 9 patients in total, including those with organic lesions and those who did not meet the requirements during data preprocessing, 63 patients were included (Figure 1). Clinical variables collected from all participants included age, years of education, patient health questionnaire‐9 (PHQ‐9), general anxiety disorder‐7 (GAD‐7), and Pittsburgh sleep quality index (PSQI). Additional clinical variables collected from patients included migraine disability assessment scale (MIDAS), disease duration (DD), visual analog scale (VAS), main pain side (left, right, and alternating sides), and average monthly headache frequency in the past 3 months (AMHF3M). According to the main pain side, patients were divided into three groups: left‐side MwoA (*n* = 22), right‐side MwoA (*n* = 14), and bilateral MwoA (*n* = 27). Among them, eight patients had chronic migraine comorbidities, with three left‐sided, two right‐sided, and three bilateral. All participants underwent routine 3D T1, QSM, and rsfMRI sequence scans, with patients being scanned within 24 h after a migraine attack.

The inclusion criteria for MwoA are as follows: (1) The diagnosis of migraine refers to MwoA in ICHD‐III 1.1; (2) age 18–50 years, right‐handed, with a headache history of more than 1 year; (3) have not used any headache preventive or treatment medication in the past month; (4) no other types of headaches besides chronic migraine, such as episodic cluster headache, medication‐overuse headache, and chronic cluster headache; and (5) no substance abuse, such as flunarizine, topiramate, valproic acid, and metoprolol.

The inclusion criteria for HC are as follows: (1) female; (2) age 18–50 years, right‐handed; (3) not pregnant or breastfeeding; (4) no history of any type of headache diagnosis or family history of migraine.

All subjects have no contraindications for MRI, history of skull trauma or surgery, hypertension, diabetes, heart disease, stroke, cognitive impairment, history of alcohol or drug abuse, other chronic systemic diseases, chronic pain, or other neurological or psychiatric disorders.

### 2.2 MRI Data Acquisition and Processing

All participants underwent MRI scans using a 3T scanner equipped with an eight‐channel head coil (MR 750W, GE Healthcare, Milwaukee, WI). The scanned sequence includes 3D T1, QSM, and rsfMRI, and the scanning parameters are as follows: (1) 3D T1: FOV = 256 × 256 × 124 mm^3^, flip angle = 12°, TR = 6.416 ms, TE = 2.16 ms, voxel size = 1 mm^3^. (2) QSM: QSM were scanned using a 3D GRE sequence with FOV = 256 × 256 × 124 mm^3^, matrix size = 256 × 256 × 124, flip angle = 20°, TR = 41.164 ms, TE_1_/spacing/TE_16_ = 3.224/2.312/37.904 ms, voxel size = 1 mm^3^ isotropic, bandwidth = 488.281 Hz/pixel, and total imaging acquisition time = 7.9 min. (3) rsfMRI: FOV = 64 × 64 × 35 mm^3^, flip angle = 90°, TR = 2000 ms, TE = 30 ms, slice thickness = 4 mm, gap = 0.6 mm, voxel size = 3.44 × 3.44 × 4.60 mm^3^, and total imaging acquisition time = 8 min.

**FIGURE 2:**
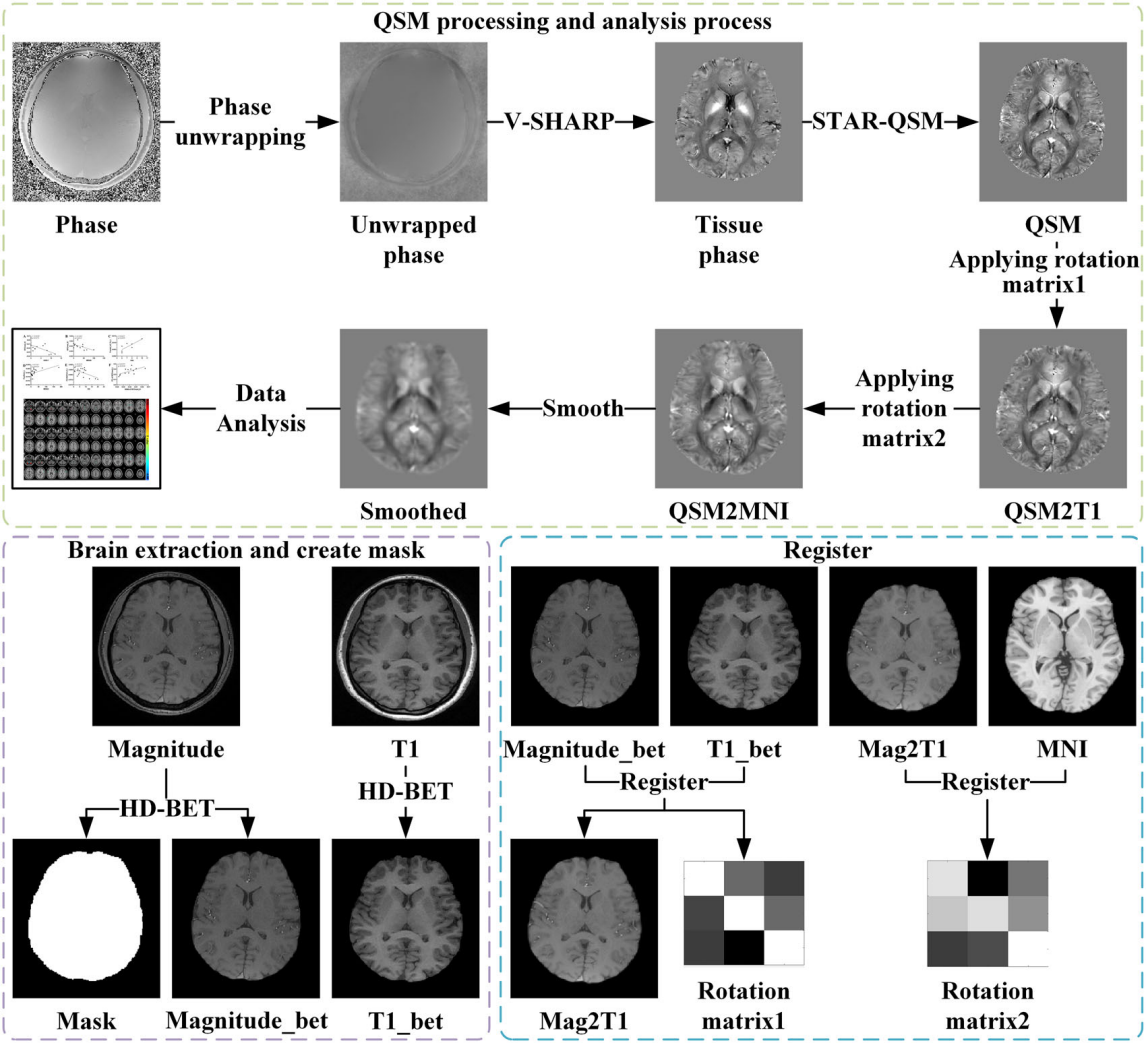
Quantitative susceptibility mapping (QSM) data processing flowchart. V‐SHARP = background field removal algorithm; STAR‐QSM = QSM inversion algorithm; QSM2T1 = QSM registration to T1; QSM2MNI = QSM registration to MNI template; HD‐BET = algorithm for extracting brain tissues and creating a brain mask; Mag2T1 = magnitude registration to T1; MNI = Montreal Neurological Institute.

QSM images were processed using the STISuite (v3.0) toolbox (https://people.eecs.berkeley.edu/~chunlei.liu/software.html), if QSM images showed large motion artifacts, they were excluded. The steps were as follows: (1) first, the brain mask was generated using HD‐BET (Isensee et al. 2019) on the first echo magnitude image; (2) then, the raw phase data was unwrapped by Laplacian‐based phase unwrapping (Schofield and Zhu 2003); (3) additionally, the V‐SHARP (Wu et al. 2012) algorithm was used to remove the background phase with a 25‐radius spherical mean filter; (4) finally, the QSM image was obtained using the STAR‐QSM (Wei et al. 2015) algorithm. Besides, the QSM image was normalized to the Montreal Neurological Institute (MNI) space using the ANTs toolbox (https://github.com/ANTsX/ANTs), as follows: (1) Using the antsRegistrationSyN.sh command to register the first echo magnitude image to T1 image to obtain the first rotation matrix, then using the antsApplyTransforms command to register QSM to T1 image by applying the first rotation matrix; (2) using the antsRegistrationSyN.sh command again to register T1 image to MNI template to obtain the second rotation matrix, then using the antsApplyTransforms command to register the result of step (1) to the MNI space by using the second rotation matrix; (3) using a Gaussian smoothing kernel with a full width at half maximum (FWHM) of 3 mm for spatial smoothing. Finally, the QSM values from all significantly different brain regions were extracted for subsequent correlation analysis. All steps are shown in Figure 2.

The preprocessing of rsfMRI was performed using the DPABI software; the main steps included as follows: (1) converting the image format to NIFTI format; (2) removing the first 10 time points; (3) time layer correction; (4) head motion correction; (5) registering to the MNI template standard space; (6) regressing out linear drift, white matter signals, cerebrospinal fluid signals, and whole brain signals; and (7) using a Gaussian smoothing kernel with an FWHM of 8 mm for spatial smoothing. If the translational motion or rotational angle of rsfMRI images exceeded 2 mm or 2°, they were excluded. The amplitudes of low‐frequency fluctuations (ALFF), fractional amplitude of low‐frequency fluctuations (fALFF), and regional homogeneity (ReHo) were calculated for all participants for a two‐sample *t*‐test. Finally, the brain regions where the peak points were located, with significant differences in the comparison results of QSM, ALFF, fALFF, and ReHo, were selected as the regions of interest (ROI) for FC analysis.

### 2.3 Statistical Analyses

The clinical variables were analyzed in SPSS 26.0 (IBM, Chicago, Illinois, USA). First, a normality test was conducted. If they were normally distributed, a one‐way analysis of variance (ANOVA) was used; otherwise, the Kruskal–Wallis test was applied. Before conducting one‐way ANOVA, a test for homogeneity of variances was performed, and the Welch test was used if unequal variances were detected. ID and FC among the four groups were analyzed using two‐sample *t*‐tests in SPM12 (http://www.fil.ion.ucl.ac.uk/spm/software/spm12). A separate two‐sample *t*‐test was performed for each pair of groups, utilizing the AAL template as a mask and considering age and years of education as covariates. Multiple comparisons were corrected using the cluster‐wise family‐wise error (FWE) rate. The QSM values from brain regions showing significant differences in ID and FC analyses were extracted, and Pearson correlation analyses were conducted with clinical variables and QSM values, as well as FC values. A significance threshold of *p* < 0.05 was used to indicate significant differences in all comparative analyses.

## 3 Results

### 3.1 Demographics and Population Characteristics

The clinical variables are listed in Table 1. The age, years of education, and PHQ‐9 among the four groups did not follow a normal distribution. The MIDAS, DD, VAS, and AMHF3M within the MwoA group did not follow a normal distribution, and the GAD‐7 had unequal variances. Significant differences existed in the years of education among the four data groups (*p* = 0.003). There were no significant differences in other clinical variables (all *p* > 0.05), including age, PHQ‐9, GAD‐7, PSQI, MIDAS, DD, VAS, and AMHF3M.

**TABLE 1:** Description of demographic and clinical variables in patients with migraine without aura (MwoA) and healthy controls.

	L (*n* = 22)	R (*n* = 14)	B (*n* = 27)	HC (*n* = 31)	*p* value
Age	31.68 ± 9.14	33.50 ± 7.82	32.78 ± 6.55	29.84 ± 7.61	0.323^a^
Education years	14.55 ± 3.58	13.71 ± 5.15	13.96 ± 3.35	16.55 ± 3.89	0.003^a^
PHQ‐9	4.73 ± 3.60	5.64 ± 5.26	7.33 ± 4.70	4.19 ± 2.61	0.069^a^
GAD‐7	4.64 ± 3.58	5.50 ± 5.76	6.44 ± 4.60	3.94 ± 2.56	0.104^b^
PSQI	8.29 ± 3.93	10.07 ± 4.75	10.11 ± 4.14	7.68 ± 3.41	0.067^c^
MIDAS	30.32 ± 35.39	46.57 ± 74.27	25.82 ± 36.34	NA	0.725^a^
DD (years)	9.27 ± 5.55	9.01 ± 6.69	8.69 ± 6.31	NA	0.826^a^
VAS	6.86 ± 1.04	6.93 ± 1.44	7.52 ± 1.67	NA	0.122^a^
AMHF3M (days/month)	8.73 ± 7.45	7.57 ± 8.49	7.22 ± 7.08	NA	0.580^a^

*Note*: Data are expressed as mean ± standard deviation. ^a^, ^b^ and ^c^ represent the *p* values for the Kruskal–Wallis test, Welch test, and one‐way ANOVA.

Abbreviations: AMHF3M = average monthly headache frequency in the past 3 months; B = bilateral migraine without aura; DD = disease duration; GAD‐7 = general anxiety disorder‐7; L = left‐side migraine without aura; MIDAS = migraine disability assessment scale; HC = healthy controls; PHQ‐9 = patient health questionnaire‐9; PSQI = Pittsburgh sleep quality index; R = right‐side migraine without aura; VAS = visual analog scale.

### 3.2 QSM Comparison

Table 2 and Supporting Information 1 reveal that, compared to HC, left‐sided MwoA exhibited significantly higher ID in the bilateral lobule VIII of the cerebellum (*p* < 0.001), left orbital inferior frontal gyrus (*p* = 0.045), left lobule VI of the cerebellum (*p* = 0.008), and left calcarine gyrus (*p* = 0.020), whereas ID in the left lobule III of the cerebellum (*p* < 0.001), right putamen (*p* = 0.003), left inferior frontal gyrus of the opercular part (*p* < 0.001), left olfactory cortex (*p* = 0.015), and left caudate nucleus (*p* = 0.016) were significantly lower. Compared to bilateral MwoA, left‐sided MwoA showed a significantly lower ID in the right olfactory cortex (*p* = 0.022). When compared to HC, MwoA (including left‐sided, right‐sided, and bilateral MwoA) performed a significant higher ID in the bilateral lobule VIII of the cerebellum (*p* < 0.001), and expressed obviously lower ID in the left lobule IX of the cerebellum (*p* < 0.001), right putamen (*p* < 0.001), left precentral gyrus (*p* = 0.031), right precentral gyrus (*p* < 0.001), right postcentral gyrus (*p* = 0.023), lobule VI of the vermis (*p* = 0.001), right lingual gyrus (*p* = 0.042), and left caudate nucleus (*p* < 0.001). No significant differences (*p* > 0.05) were observed in comparisons between other groups, including left‐sided MwoA versus right‐sided MwoA, right‐sided MwoA versus HC, right‐sided MwoA versus bilateral MwoA, and bilateral MwoA versus HC.

**TABLE 2:** Comparison of quantitative susceptibility mapping (QSM) on different headache sides.

	Anatomic region (AAL3v1)	Cluster size	Peak coordinate in MNI	*t* value	*p* value
L vs. HC	Cerebellum_8_L	1115	−8, −69, −34	5.78	< 0.001
	Cerebellum_8_R	1863	32, −63, −57	5.12	< 0.001
	Frontal_Inf_Orb_2_L	365	−37, 34, −7	4.77	0.045
	Cerebellum_6_L	505	−36, −46, −27	4.50	0.008
	Calcarine_L	427	−16, −66, 15	4.17	0.020
	Cerebellum_3_L	973	−3, −43, −21	−5.98	< 0.001
	Putamen_R	596	27, 9, 1	−5.48	0.003
	Frontal_Inf_Oper_L	818	−54, 8, 21	−5.21	< 0.001
	Olfactory_L	451	−3, 19, −10	−4.67	0.015
	Caudate_L	446	−8, 8, 0	−4.33	0.016
L vs. B	Olfactory_R	406	4, 19, −9	−4.38	0.022
MwoA vs. HC	Cerebellum_8_R	3558	29, −64, −57	6.00	< 0.001
	Cerebellum_8_L	3308	−32, −67, −58	5.23	< 0.001
	Cerebellum_9_L	1263	−8, −55, −57	−6.61	< 0.001
	Putamen_R	1000	27, 9, 1	−5.01	< 0.001
	Precentral_L	422	−59, 10, 38	−4.83	0.031
	Precentral_R	1365	60, 10, 27	−4.74	< 0.001
	Postcentral_R	448	61, −5, 38	−4.66	0.023
	Vermis_6	766	3, −58, −21	−4.42	0.001
	Lingual_R	396	12, −75, −12	−4.40	0.042
	Caudate_L	827	−9, 9, 1	−4.31	< 0.001

Abbreviations: B = bilateral migraine without aura; L = left‐side migraine without aura; MNI = Montreal Neurological Institute; MwoA = migraine without aura, including left‐sided, right‐sided, and bilateral groups; HC = healthy controls; R = right‐side migraine without aura.

### 3.3 Functional Connectivity

Compared to HC, left‐sided MwoA demonstrated significantly higher fALFF values in the left precentral gyrus (*p* = 0.010) and lower ReHo values in the left superior temporal gyrus of the temporal pole (*p* = 0.003). When compared to right‐sided MwoA, left‐sided MwoA exhibited significantly lower ALFF values in the right pregenual of the anterior cingulate cortex (*p* = 0.006). Compared to bilateral MwoA, left‐sided MwoA showed apparently lower ALFF values in the right anterior orbital gyrus (*p* = 0.001) and lower fALFF values in the right medial orbital gyrus (*p* = 0.004) (Table 3). No significant differences (*p* > 0.05) were observed in comparisons among other groups in ALFF, fALFF, and ReHo, including right‐sided MwoA versus HC, right‐sided MwoA versus bilateral MwoA, bilateral MwoA versus HC, and MwoA (including left‐sided, right‐sided, and bilateral MwoA) versus HC.

**TABLE 3:** Comparison of amplitude of low‐frequency fluctuations (ALFF), fractional amplitude of low‐frequency fluctuations (fALFF), and regional homogeneity (ReHo) among different sides of headache.

		Anatomic region (AAL3v1)	Cluster size	Peak coordinate in MNI	*t* value	*p* value
L vs. HC	ALFF	Precentral_L	85	−33, −21, 72	4.78	0.010
	ReHo	Temporal_Pole_Sup_L	133	−27, 9, −21	−5.24	0.003
L vs. R	ALFF	ACC_pre_R	91	3, 45, 24	−5.35	0.006
L vs. B	ALFF	OFCant_R	136	21, 36, −18	−4.66	0.001
	fALFF	OFCmed_R	80	18, 42, −24	−6.38	0.004

Abbreviations: B = bilateral migraine without aura; L = left‐side migraine without aura; MNI = Montreal Neurological Institute; HC = healthy controls; R = right‐side migraine without aura.

**FIGURE 3:**
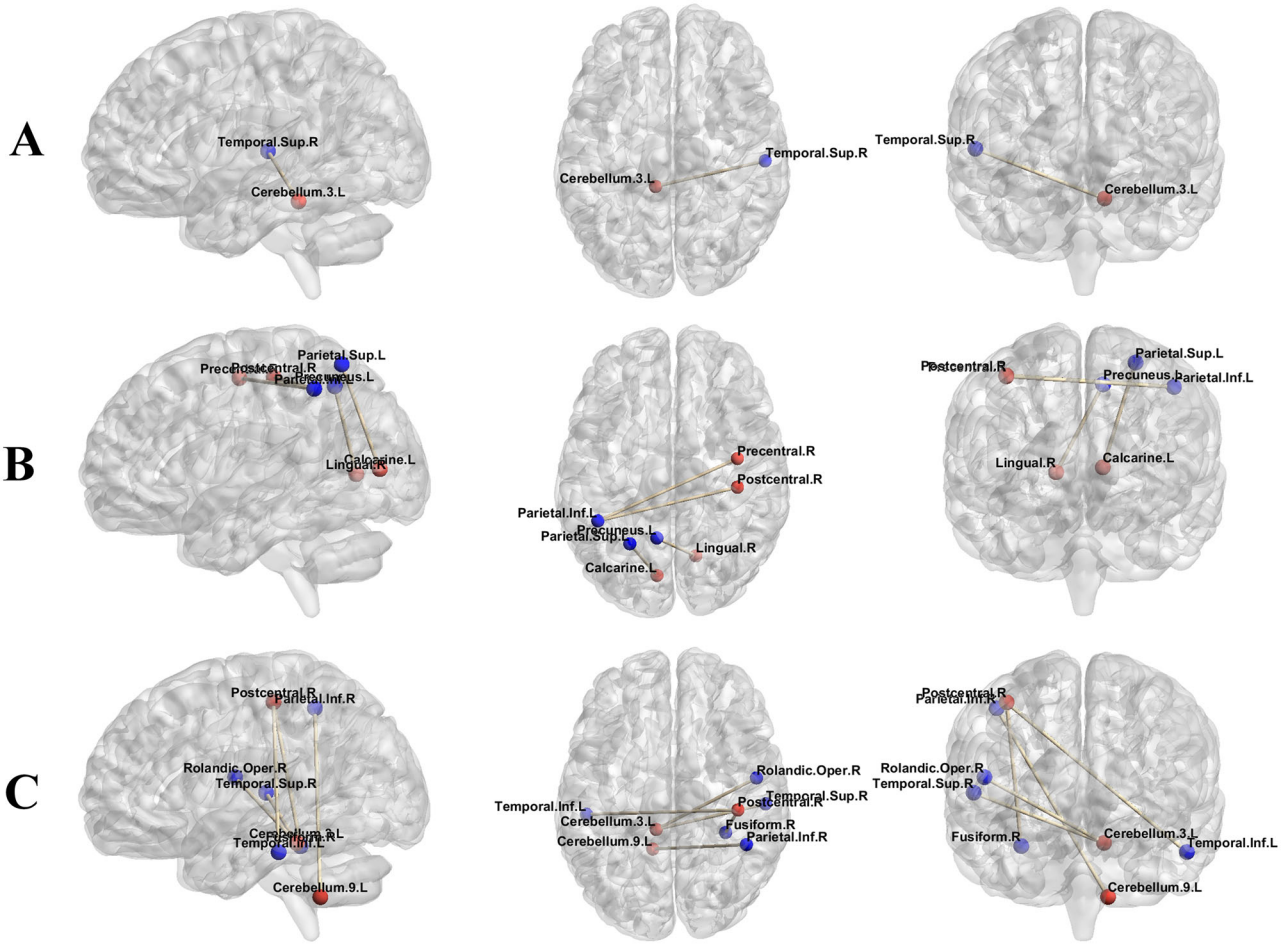
Functional connectivity (FC) diagram between subgroups of migraine without aura (MwoA). Part (A) represents the comparison results of FC between left‐sided MwoA and healthy controls (HC); part (B) represents the FC comparison results between left‐sided MwoA and bilateral MwoA; part (C) represents the FC comparison results between MwoA (including left‐sided, right‐sided, and bilateral MwoA) and HC. The red nodes represent regions of interest. However, the blue nodes represent brain regions with FC differences from the regions of interest.

The results of FC analysis showed that compared to HC, left‐sided MwoA exhibited significantly enhanced FC between the left lobule III of the cerebellum and the right superior temporal gyrus (*p* = 0.002) (Figure 3). Compared to bilateral MwoA, the FC of left‐sided MwoA was obviously enhanced between the left calcarine gyrus and the left superior parietal gyrus (*p* = 0.004), the right precentral gyrus and the left inferior parietal gyrus (*p* = 0.001), the right postcentral gyrus and the left inferior parietal gyrus (*p* < 0.001), and the right lingual gyrus and the left precuneus (*p* = 0.021). Upon comparing MwoA (including left‐sided, right‐sided, and bilateral MwoA) and HC, we found that the FC of MwoA had enhanced between the left lobule III of the cerebellum and both the left superior temporal gyrus (*p* < 0.001) and the right Rolandic operculum (*p* = 0.010); between the left lobule IX of the cerebellum and the right inferior parietal (*p* = 0.047); between the right postcentral gyrus and both the left inferior temporal gyrus (*p* = 0.016) and the right fusiform gyrus (*p* = 0.018) (Table 4). There were no significant statistical differences (*p* > 0.05) in the FC analysis of other ROI, including the bilateral lobule VIII of the cerebellum, the left lobule VI of the cerebellum, the right putamen, and the left caudate nucleus.

**TABLE 4:** Results of functional connectivity (FC).

	ROI	Anatomic region (AAL3v1)	Cluster size	Peak coordinate in MNI	*t* value	*p* value
L vs. HC	Cerebellum_3_L	Temporal_Sup_R	301	54, −9, −12	4.85	0.002
L vs. B	Calcarine_L	Parietal_Sup_L	243	−24, −69, 51	4.87	0.004
	Precentral_R	Parietal_Inf_L	345	−39, −45, 51	4.85	0.001
	Postcentral_R	Parietal_Inf_L	417	−36, −48, 51	4.73	< 0.001
	Lingual_R	Precuneus_L	160	−6, −57, 69	4.79	0.021
MwoA vs. HC	Cerebellum_3_L	Temporal_Sup_L	598	−57, −3, −6	4.48	< 0.001
		Rolandic_Oper_R	228	42, −15, 18	4.25	0.010
	Cerebellum_9_L	Parietal_Inf_R	138	51, −48, 45	4.28	0.047
	Postcentral_R	Temporal_Inf_L	196	−42, −63, −9	4.19	0.016
		Fusiform_R	189	30, −66, −6	4.19	0.018

Abbreviations: B = bilateral migraine without aura; L = left‐side migraine without aura; MNI = Montreal Neurological Institute; MwoA = migraine without aura; ROI = regions of interest; HC = healthy contols.

### 3.4 Correlation Analysis

**FIGURE 4:**
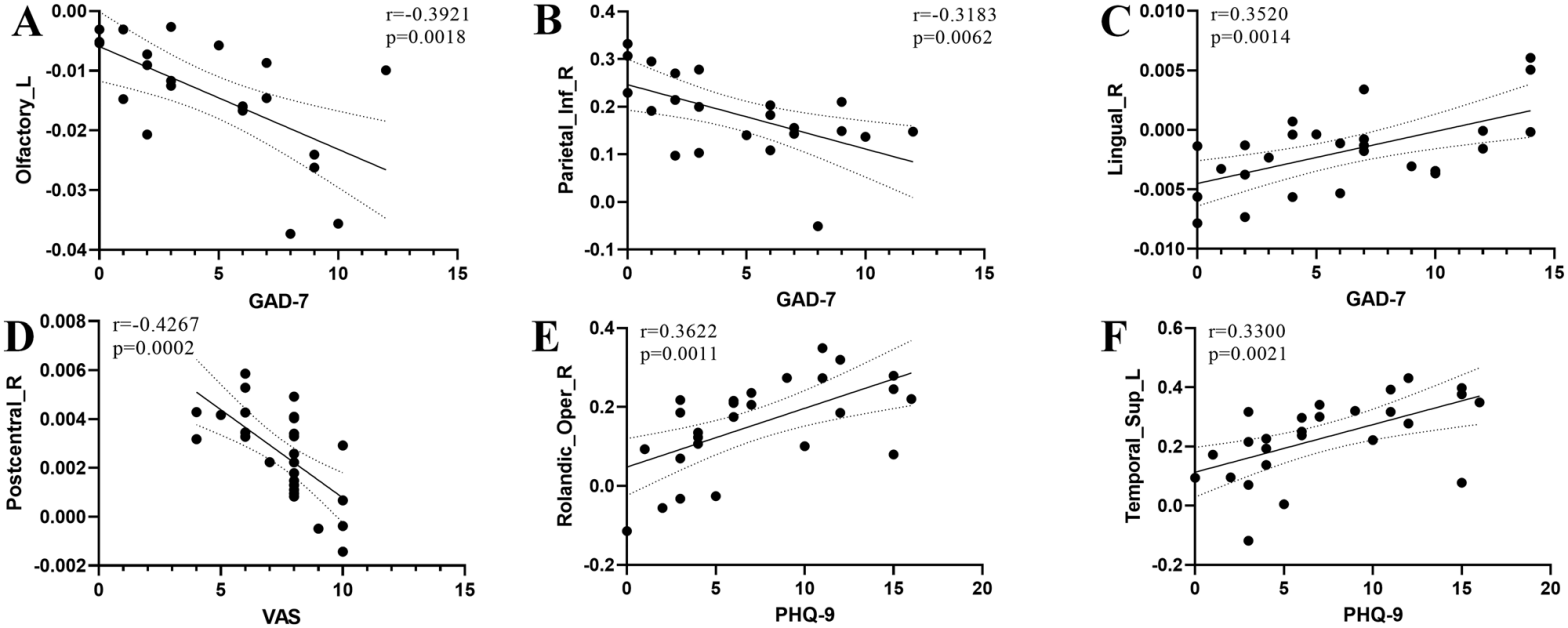
The correlation between clinical variables and both quantitative susceptibility mapping (QSM) and functional connectivity values. The dotted line represents the 95% confidence interval. (A and B) The QSM values of the left olfactory cortex and the right inferior parietal gyrus exhibited prominent negative correlations with GAD‐7 in left‐sided migraine without aura (MwoA); (C) the QSM values of the right lingual gyrus expressed noticeable positive correlations with GAD‐7 in bilateral MwoA; (D) the QMS values of the right postcentral gyrus showed a significant negative correlation with VAS in bilateral MwoA; (E and F) the functional connectivity values of between the left lobule III of the cerebellum and both the left superior temporal gyrus and the right Rolandic operculum had emphatic positive correlations with PHQ‐9 in bilateral MwoA. GAD‐7 = general anxiety disorder‐7; PHQ‐9 = patient health questionnaire‐9; VAS = visual analog scale.

The results are shown in Figure 4. It was found that among left‐sided MwoA, the QSM values of the left olfactory cortex (*r* = −0.3921, *p* = 0.0018) and the right inferior parietal gyrus (*r* = −0.3183, *p* = 0.0062) exhibited prominent negative correlations with GAD‐7. In bilateral MwoA, the QSM values of the right lingual gyrus expressed noticeable positive correlations with GAD‐7 (*r* = 0.3520, *p* = 0.0014). The QSM values of the right postcentral gyrus showed a significant negative correlation with VAS (*r* = −0.4267, *p* = 0.0002). Additionally, the FC values between the left lobule III of the cerebellum and both the left superior temporal gyrus (*r* = 0.3622, *p* = 0.0011) and the right Rolandic operculum (*r* = 0.3300, *p* = 0.0021) had emphatic positive correlations with PHQ‐9. No significant correlations (*p* > 0.05) were found between other clinical variables and both QSM values and FC values, including PSQI, MIDAS, DD, and AMHF3M. And no significant correlations (*p* > 0.05) were found in other groups, including HC, right‐sided MwoA, and MwoA (including left‐sided, right‐sided, and bilateral MwoA).

## 4 Discussion

In this study, we conducted ID and FC analyses using rsfMRI and QSM data in 63 female patients with MwoA (22 on left‐side MwoA, 14 on right‐side MwoA, and 27 on bilateral MwoA) and 31 matched HC. Our findings indicated that there were significant differences in ID and FC among MwoA subgroups with different main pain sides. Furthermore, the correlation between clinical variables and both ID and FC in brain regions also showed significant differences among the MwoA subgroups.

Current research indicates that intracellular iron accumulation plays a crucial role in the occurrence and development of migraines (Goadsby 2012; Tepper et al. 2012; Kruit et al. 2009). Iron in the brain is primarily stored in ferritin and transported by transferrin. Studies have shown that migraines significantly increased the expression of iron storage proteins in microglia and meningeal cells (Saletta et al. 2011), leading to abnormal expression of iron transport and storage proteins, which, in turn, significantly increased intracellular iron accumulation. Furthermore, the central nervous system interacts with capillaries through the end‐feet of astrocytes, which are part of the blood–brain barrier structure (Nedergaard, Ransom, and Goldman 2003) and could directly circulate excess iron (Zarruk et al. 2015), resulting in abnormal iron accumulation in the brain tissue of migraine patients. Research found elevated total oxidant levels and oxidative stress indices in MwoA patients (Jiménez‐Jiménez et al. 2024). Oxidative stress may have damaged neurons in migraine patients, causing damaged neurons to release iron, thus increasing ID in the brain. Excessive brain iron could lead to abnormal FC between brain networks. Abnormal brain iron may cause oxidative stress reactions and glial cell dysfunction (Galaris, Barbouti, and Pantopoulos 2019; McCarthy and Kosman 2015), which, in turn, damaged neurons, impairing communication between neurons and reducing the stability and efficiency of brain FC. This aligns with our findings (Tables 2 and 4).

Previous studies reported higher ID in the putamen of migraine patients. For example, compared with healthy volunteers, migraine patients under the age of 50 (including those with and without aura) had higher iron content in the putamen (Kruit et al. 2009), and patients with episodic migraines had higher magnetic susceptibility values in the left putamen (Li et al. 2024). Additionally, chronic migraine patients showed higher ID in the left precentral gyrus (Chen et al. 2022) and bilateral postcentral gyrus (Chen et al. 2021) compared to HC. However, in our study, female patients with MwoA had lower ID in the left putamen, bilateral precentral gyrus, and right postcentral gyrus compared to HC. The main differences from these studies were as follows: (1) The study subjects were different as we included only female patients; (2) the headache types were different as we only studied patients with MwoA; (3) the number and quality of patients were different as we had a larger sample size and ensured MRI scans were conducted within 24 h after onset. We believe the lower ID in these brain regions might have been due to (1) during migraine attacks, inflammatory mediators cause dysfunction of transferrin and its receptors (Rouault 2006; Ganz 2011), hindering iron from entering brain tissue; (2) iron is used to synthesize neurotransmitters related to depression or anxiety during migraine attacks (Southwick et al. 1999; Hamon and Blier 2013), increasing the demand for iron; (3) neurons are damaged after migraine attacks, and cell repair and regeneration required iron (Saletta et al. 2011; Lukianova and David 2005), leading to increased iron consumption.

We found that differences in both ID and FC in left‐sided MwoA patients primarily occur in the left hemisphere, likely related to (Chen et al. 2022) the pain modulation circuit and cognitive control functions of the left hemisphere. Surprisingly, patients with left‐sided MwoA exhibited differences in ID in the right lobule VIII of the cerebellum and the right putamen, and these two brain regions are related to pain sensation (Starr et al. 2011; Ruscheweyh et al. 2014). We think this may be related to the complex neural networks and functional connections in the brain, where pain signals could propagate through neural pathways and FC networks to the contralateral side of the brain (Fox and Raichle 2007), activating corresponding brain regions.

We observed more severe damage in the cerebellum of patients with left‐sided MwoA (Tables 2 and 4). The cerebellum plays a crucial role in pain processing, having connections with multiple cortical areas. Studies indicate that the structure and function of the cerebellum are altered in migraine patients (Mehnert and May 2019). Researchers have identified cerebellar circuits involved in motor control and cognitive functions (Mannarelli et al. 2023). It has been confirmed that cognitive and executive function impairments exist during migraine attacks (Santangelo et al. 2016). Our research results suggest that the cerebellar dysfunction in patients with left‐sided MwoA may be related to the damaging stimuli and the cerebellum's involvement in processing and modulating pain caused by frequent migraine attacks.

We found significant differences in ID, FC, brain neural activity (Table 3), and correlation analyses among MwoA subgroups with different primary headache sides. Wang et al. (2023) suggested further subclassifying MwoA patients into subgroups, whereas Blum et al. (2023) reported differences between left‐ and right‐sided migraine patients across multiple domains. A recent study further divided migraine into left‐ and right‐sided migraine (Sprouse Blum et al. 2024), attempting to clarify whether differences exist between them and found that left‐sided migraine patients reported higher headache frequency and severity than right‐sided migraine patients. Our experimental results indicated that differences between subgroups of MwoA are manifested in brain regions such as the olfactory cortex, parietal lobe, frontal lobe, anterior cingulate gyrus, and precuneus, which have been confirmed in previous migraine studies (Hu et al. 2023). Previous studies have reported some correlations between clinical variables and FC or ID in MwoA patients: FC between the dorsolateral prefrontal cortex and the orbitofrontal cortex is positively correlated with migraine duration (Jin et al. 2013); short‐range FC density between the left prefrontal cortex, putamen, and caudate nucleus is significantly negatively correlated with the DD (Gao et al. 2016). In comparison to the previous research, our study results indicated a different correlation between clinical variables and both ID and FC, affected by migraine laterality (Figure 4). These findings may explain why different studies have reported different differing brain regions, as the number of MwoA subgroups varies.

The limitations of our research include as follows: (1) There were significant differences in the years of education among different groups in clinical variables, but we included them as covariates in the two‐sample *t*‐tests. This method aims to control for potential confounding effects and ensure that any differences observed in the primary variable are not solely attributed to clinical factors. (2) Limited data on patients with right‐side migraines may lead to a lack of broad representativeness in the analysis results. Additionally, the study data were derived from a relatively homogeneous center. In the future, we will conduct more comprehensive research by combining data from multiple centers. (3) Our study focused on female migraine patients without warning signs, which is not yet clear whether male patients have similar results.

## 5 Conclusion

We categorized female MwoA patients into different subgroups based on the primary pain side and conducted analyses of ID, FC, and correlations. Preliminary evidence indicates significant differences in ID, FC, and correlations among MwoA subgroups. This provides neuroimaging references for further subclassifying MwoA patients. This may explain why different studies have reported different differing brain regions, providing valuable insights into the potential pathophysiological mechanisms underlying brain functional impairments in women with MwoA, providing a reference for comprehensive understanding and subsequent research.

## Author Contributions

**Yan Zhang**: writing–original draft, software; visualization, methodology, data curation. **Mingxian Bai**: methodology, conceptualization, formal analysis. **Zhenliang Xiong**: software, validation. **Qin Zhang**: investigation, formal analysis, data curation, conceptualization. **Lihui Wang**: funding acquisition, project administration. **Xianchun Zeng**: project administration, funding acquisition, writing–review and editing, resources, supervision.

## Ethics Statement

The study complies with the statement published in the World Medical Association Declaration of Helsinki. The hospital ethics committee approved this study [Ethics Review (Scientific Research) No. 2023‐081].

## Consent

All participants signed a written informed consent form.

### Peer Review

The peer review history for this article is available at https://publons.com/publon/10.1002/brb3.70096.

## Supporting information



Supporting Information

## Data Availability

The datasets analyzed or generated during the present study are available from the corresponding author on request.
