# Supplementary figures and images for: Iron Deposition and Functional Connectivity Differences in Females With Migraine Without Aura: A Comparative Study of Headache Sides
Source: Brain Behav. 2024 Oct 22;14(10):e70096. doi: 10.1002/brb3.70096 (PMC11494401; doi:10.1002/brb3.70096)

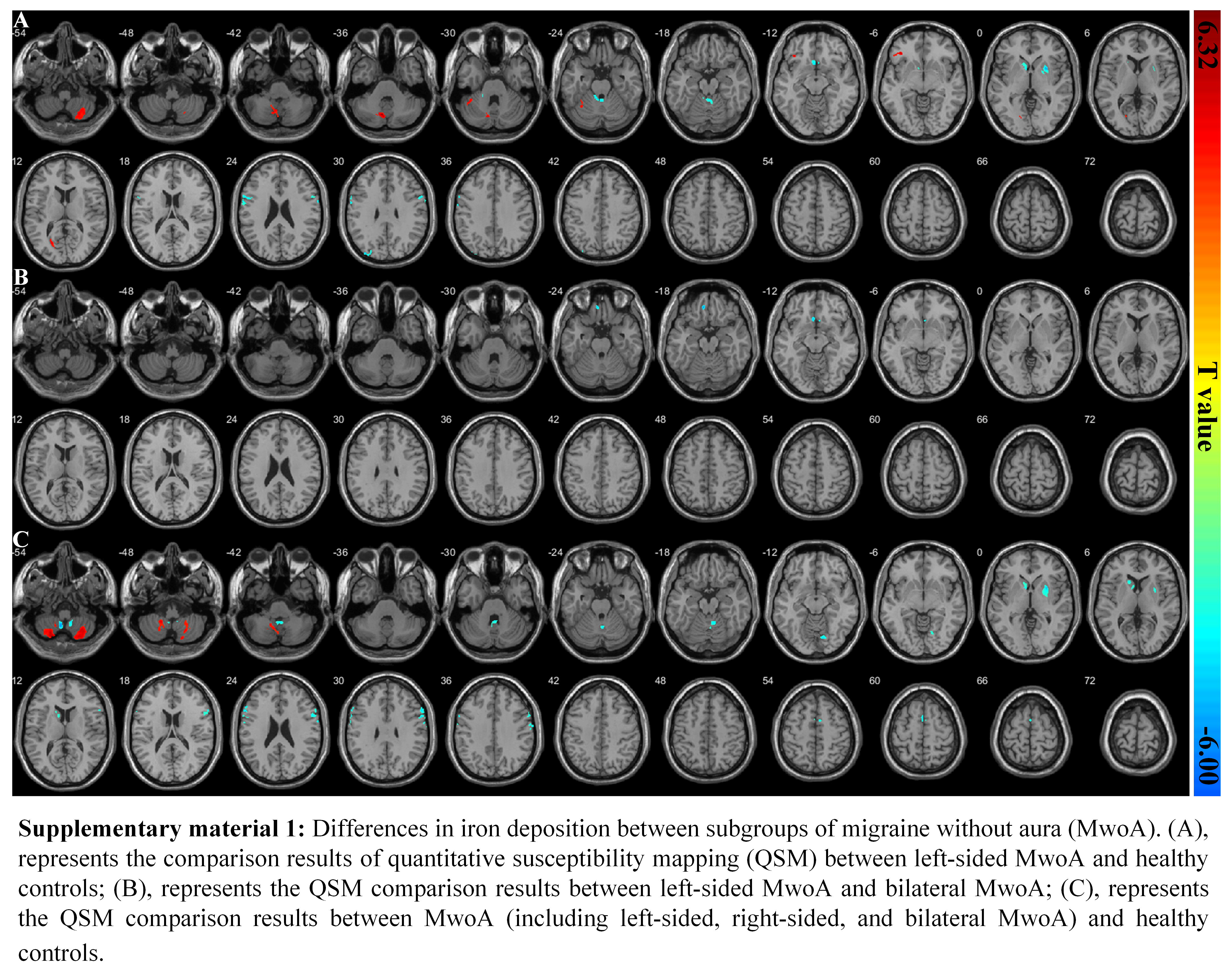

Supplement: Supplementary file 1 — Supporting Information [file BRB3-14-e70096-s001.png]
